# Discovery of a Potent RIPK3 Inhibitor for the Amelioration of Necroptosis-Associated Inflammatory Injury

**DOI:** 10.3389/fcell.2020.606119

**Published:** 2020-12-08

**Authors:** Kaijiang Xia, Fang Zhu, Chengkui Yang, Shuwei Wu, Yu Lin, Haikuo Ma, Xiaoliang Yu, Cong Zhao, Yuting Ji, Wenxiang Ge, Jingrui Wang, Yayun Du, Wei Zhang, Tao Yang, Xiaohu Zhang, Sudan He

**Affiliations:** ^1^Jiangsu Key Laboratory of Neuropsychiatric Diseases and College of Pharmaceutical Sciences, Soochow University, Suzhou, China; ^2^Center of Systems Medicine, Institute of Basic Medical Sciences, Chinese Academy of Medical Sciences & Peking Union Medial College, Beijing, China; ^3^Suzhou Institute of Systems Medicine, Suzhou, China; ^4^Key Laboratory of Synthetic Biology Regulatory Elements, Chinese Academy of Medical Sciences, Beijing, China; ^5^Cyrus Tang Hematology Center and Collaborative Innovation Center of Hematology, State Key Laboratory of Radiation Medicine and Protection, Soochow University, Suzhou, China; ^6^School of Life Sciences and Technology, China Pharmaceutical University, Nanjing, China

**Keywords:** necroptosis, RIPK3, kinase inhibitor, inflammatory diseases, Zharp-99

## Abstract

Necroptosis is a form of regulated necrosis that requires the activation of receptor-interacting kinase 3 (RIPK3 or RIP3) and its phosphorylation of the substrate MLKL (mixed lineage kinase domain-like protein). Necroptosis has emerged as important cell death involved in the pathogenesis of various diseases including inflammatory diseases, degenerative diseases, and cancer. Here, we discovered a small molecule Zharp-99 as a potent inhibitor of necroptosis through blocking the kinase activity of RIPK3. Zharp-99 efficiently blocks necroptosis induced by ligands of the death receptor and Toll-like receptor as well as viral infection in human, rat and mouse cells. Zharp-99 strongly inhibits cellular activation of RIPK3, and MLKL upon necroptosis stimuli. Zharp-99 directly blocks the kinase activity of RIPK3 without affecting RIPK1 kinase activity at the tested concentration. Importantly, Zharp-99 exerts effective protection against TNF-α induced systemic inflammatory response syndrome in the mouse model. Zharp-99 displays favorable *in vitro* safety profiles and *in vivo* pharmacokinetic parameters. Thus, our study demonstrates Zharp-99 as a potent inhibitor of RIPK3 kinase and also highlights its potential for further development of new approaches for treating necroptosis-associated inflammatory disorders.

## Introduction

Necroptosis is a form of regulated cell death that shows necrotic features including cell swelling and disrupted cell membrane. Necroptosis is tightly regulated by the activation of receptor-interacting protein kinase 1 (RIPK1 or RIP1) and RIPK3 (RIP3; Linkermann and Green, 2014; He and Wang, 2018; Mifflin et al., 2020). In TNF-induced necroptosis, RIPK1 interacts with RIPK3 through their RIP homotypic interaction motif (RHIM) domains, leading to RIPK3 activation and phosphorylation (Holler et al., 2000; Cho et al., 2009; He et al., 2009; Zhang et al., 2009). Active RIPK3 phosphorylates its substrate mixed lineage kinase domain-like protein (MLKL; Sun et al., 2012; Zhao et al., 2012). The phosphorylation of MLKL results in MLKL oligomerization and membrane translocation to mediate cell rupture (Murphy et al., 2013; Cai et al., 2014; Chen et al., 2014; Wang et al., 2014a). As a lytic cell death, necroptosis elicits inflammatory responses via the release of cellular contents including damage-associated molecular patterns (DAMPs; Linkermann and Green, 2014; He and Wang, 2018; Mifflin et al., 2020). Necroptosis plays important roles in a variety of pathological conditions including inflammatory disorders, ischemia-reperfusion-induced injury, degenerative diseases and cancer. Thus, strategies to interfere with the necroptosis signaling pathway could be potentially developed for the treatment of necroptosis-related diseases.

Receptor-interacting kinase 3 has emerged as a key molecule of necroptosis that can be initiated by various signals including activation of death receptors, Toll-tike receptors, interferon receptors as well as pathogen infection3 (Linkermann and Green, 2014; He and Wang, 2018; Mifflin et al., 2020). Receptor-interacting kinase 3 contains an N-terminal serine/threonine kinase domain and a C-terminal RHIM domain. The kinase activity of RIPK3 is essential for its activation and phosphorylation of MLKL (Cho et al., 2009; He et al., 2009; Sun et al., 2012; Zhang et al., 2009; Zhao et al., 2012). Increasing evidence suggests that RIPK3 can be activated for necroptosis by other RHIM-containing proteins including ZBP1/DAI/DLM1 (Z-nucleic acid binding protein) (Upton et al., 2012; Samir et al., 2020)and TRIF/TICAM-1 (Toll/IL-1 receptor domain-containing adaptor inducing IFN-β) (He et al., 2011; Kaiser et al., 2013) in addition to RIPK1. Thus, necroptosis can proceed independent of RIPK1, leading to the assumption that RIPK3 may protect cell from a broader range of necroptotic pathologies. The combination of tractability and broad dedicated role in necroptosis makes RIPK3 an attractive target for modulating necroptosis and its related diseases.

Considering the essential role of RIPK3 kinase activity in necroptosis, the kinase domain of RIPK3 is an interesting target for intervention with small molecule inhibitors. A number of RIPK3 inhibitors have been reported including the FDA approved drugs dabrafenib, sorafenib, and ponatinib (Kaiser et al., 2013; Li et al., 2014; Mandal et al., 2014; Fauster et al., 2015; Park et al., 2018; Zhang et al., 2019; Hart et al., 2020). Inhibition of RIPK3 was discovered as an off-target effect of these drugs as opposed to the intended targets such as Braf, VEGRF, and Bcr-Abl (Fauster et al., 2015; Li et al., 2014). The classical work on GSK’840, GSK’843, GSK’872 established RIPK3 as a potential drug target with a caveat: inhibition of RIPK3 kinase activity induced apoptosis in a concentration-dependent manner (Kaiser et al., 2013; Mandal et al., 2014). The interaction of compound with RIPK3 imposed a conformation change which drove the recruitment of RIPK1 via the RHIM domain and activated caspase 8 for the initiation of apoptosis (Mandal et al., 2014). It is not clear what structural features of a compound may avoid the induction of apoptotic RIPK3 conformational change. Reflecting these challenges, there is no RIPK3 inhibitor currently under clinical investigation. We have a long standing interest in studying RIPK3 and its role in necroptosis. Our ultimate goal is to identify novel RIPK3 inhibitors with high efficacy and low toxicity. Here, we wish to report the discovery of Zharp-99 as a novel inhibitor of RIPK3 kinase activity. Zharp-99 exhibits potent cellular efficacy of inhibiting necroptosis induced by multiple necroptotic stimuli in human, mouse, and rat cells. Zharp-99 displays favorable in vitro safety profiles and in vivo pharmacokinetic parameters. Importantly, pre-treatment of Zharp-99 significantly ameliorates TNF-induced systemic inflammatory response syndrome (SIRS) in the mouse model. These findings highlight Zharp-99 as a potent RIPK3 inhibitor and suggest the potential of Zharp-99 as a starting point for the development of new approaches to treat necroptosis-associated disorders.

## Materials and Methods

### Cell Culture

Human colon cancer HT-29 and mouse fibrosarcoma L929 cells were from ATCC. Mouse embryonic fibroblasts (MEF), HT-29 cells stably expressing RIPK3-shRNA and shRNA resistant scramble Flag-tagged RIPK3 (W46), and NIH3T3 cell line stably expressing RIPK3 fused to mutant FK506-binding protein (NIH3T3-RIPK3) were kindly provided by Dr. Xiaodong Wang [National Institute of Biological Sciences (NIBS), Beijing]. HeLa-MLKL (1-190) cell line was a gift from Dr. Zhigao Wang (University of Texas Southwestern Medical Center at Dallas). These cells were cultured in Dulbecco’s modified Eagle’s medium (Hyclone) supplemeted with 10% fetal bovine serum (Invitrogen) and 2 mM L-glutamine (Invitrogen) in a humidified incubator at 37°C and 5% CO_2_. NIH3T3-RIPK3 was cultured in complete medium containing 2 μg/ml G418 (Calbiochem). HeLa-MLKL (1–190) stable line were cultured in complete medium containing 10 μg/ml Blasticidin plus 1 μg/ml puromycin. Bone marrow-derived macrophages were isolated from the bone marrow of 6–8 week old mice and rats, and cultured for 7 days in the medium containing 30% L929-cell conditioned medium, 20% FBS, and 50% RPMI-1640. L929 cell conditioned medium containing colony stimulating factor was collected after growing L929 cells in DMEM plus 10% FBS for 7 to 10 days previously described (He et al., 2011).

### Cell Viability Assay

Cells were seeded in 96-well plates and then treated as indicated. The cell viability was determined by assessment of ATP levels using the CellTiter-Glo Luminescent Cell Viability Assay kit following the manufacture’s instructions (Promega). Luminescence was calculated with SpectraMax i3x (Molecular Devices).

### Reagents and Antibodies

Human TNF-α recombinant protein was generated as previously described (Wang et al., 2008). Mouse TNF-α recombinant protein was purchased from Genscript. The Smac mimetic compound and anti-human RIPK3 antibody were kindly provided by Dr. Xiaodong Wang (National Institute of Biological Sciences, Beijing). z-VAD was purchased from Bachem respectively. Lipopolysaccharide (LPS) was purchased from Sigma. Mouse recombinant RIPK1 and RIPK3 were purchased from SignalChem. The cellTiter-Glo Luminescent cell viability assay kit and ADP-Glo kinase assay kit were purchased from Promega. The following antibodies were used: RIPK1 (BD Biosciences, 610458), p-hRIPK1 (CST, 65746), p-hRIPK3 (Abcam, 209384), hMLKL (Abcam, 184718), p-hMLKL (Abcam, 187091), p-mRIPK1 (CST, 31122S), mRIPK3 (Prosci, 2283), p-mRIPK3 (CST, 91702), mMLKL (Abgent, 14272b), p-mMLKL (Abcam, 196436), β-actin (Sigma, A2066). Mouse IL-6 ELISA kit was from MultiSciences (Lianke).

### Western Blot Analysis

The cell pellets were harvested and dissolved in lysis buffer (20 mM Tris–HCl, pH 7.4 150 mM NaCl, 1% Triton X-100, 1 mM Na_3_VO_4_, 10% glycerol, 25 mM β-glycerol-phosphate, 0.1 mM PMSF, with the Sigma phosphatase inhibitors and the Roche Pierce protease inhibitor set). The re-suspended cell pellet was then incubated on ice for 20 min, followed by centrifugation at 13000 × *g* for 20 min at 4°C. The supernatants were collected and protein concentrations were measured using the BCA Protein Assay Kit (Thermo Fisher Scientifc, United States). Finally, cell lysates were subjected for western-blot analysis of the indicated antibodies.

### RNA Extraction and Real-Time PCR

Total RNA was extracted using TRIzol Reagent (Invitrogen) from cell pellets. cDNA was synthesized using a Revert Aid First Strand cDNA kit (Thermo Fisher Scientifc). Real-time PCR was performed using corresponding primers and Power SYBR^®^ Green PCR Master Mix (Invitrogen). The data were normalized to GAPDH. The primer sequences are as follows: ICP6 Forward: GGCTGCAATCGGCCCTGAAGTA, Reverse: GGT GGTCGTAGAGGCGGTGGAA; TNFα Forward: CCCTCACAC TCAGATCATCTTCT, Reverse: GCTACGACGTGGGCTACAG; CCL3 Forward: TTCTCTGTACCATGACACTCTGC, Reverse: CGTGGAATCTTCCGGCTGTAG; CXCL1 Forward: GCACCC AAACCGAAGTCATAG, Reverse: AGAAGCCAGCGTTCAC CAGA; GAPDH Forward: CAAGAAGGTGGTGAAGCAGGC, Reverse: CATACCAGGAAATGAGCTTGAC.

### *In vitro* Kinase Activity Assay

The recombinant human RIPK1 or RIPK3 protein was incubated with the control DMSO or the indicated compound for around 15 min in the assay buffer (25 mM HEPES PH7.2, 12.5 mM MnCl_2_, 5 mM EGTA, 20 mM MgCl_2_, 12.5 mM β-glycerol phosphate, 2 mM EDTA, and 2 mM DTT). ATP (50 μM) and the substrate MBP (20 μM) were then added to the reaction at room temperature for 2 h. The kinase activity was calculated by the measuring the luminescence after the addition of the ADP-Glo Kinase Assay kit according to the manufacture’s instructions (Promega).

### Source of Animals

C57BL/6 male mice were purchased from Suzhou JOINN Clinical Co., Ltd. All male mice were bred under standard conditions and used at the age of 6–7 weeks with about 18–20 g body weight. All animal experiments were performed in accordance with protocols approved by the Institutional Animal Care and Use Committee at Suzhou Institute of Systems Medicine.

### TNF-Induced Systemic Inflammatory Response Syndrome

Zharp-99 was diluted into sterile PBS containing 40% PEG400. C57BL/6 mice were pretreated with vehicle or Zharp-99 (5 mg/kg) via intraperitoneal injection for around 15 min, followed by the administration of mouse TNF-α (6.5 μg/mouse) via tail intravenous injection. The status of mice was monitored by measuring anal temperature. Mice mortality was continuously monitored till 60 h after TNF-α administration. Blood was collected 4 h post TNF-α challenge and serum was isolated for further examination.

### Methods for Determination of CYP/hERG Inhibition and Pharmacokinetic Parameters

Cytochrome P450 (CYP) inhibitory potency was determined by industrial standard methods as reported previously (Dong et al., 2015). Methods to determine potassium channel hERG inhibition and in vivo pharmacokinetic parameters have been previously published (Lu et al., 2017).

### Synthesis of Zharp-99

The synthetic scheme and detailed experimental procedure as well as spectroscopic characterizations of Zharp-99 can be found in the supporting information.

### Statistical Analyses

Data of cell survival rate are represented as the mean ± standard deviation of duplicates or triplicates from one representative experiment (*n* ≥ 2 independent experiments). Significance was analyzed using *t*-tests of GraphPad Prism software. *P*-values were defined by ONE-way ANOVA and multi-comparison test for statistics analysis. ^∗^*P* < 0.05, ^∗∗^*P* < 0.01, ^∗∗∗^*P* < 0.001.

## Results

### Zharp-99 Efficiently Blocks TNF-Induced Necroptosis in Both Human and Mouse Cells

**FIGURE 1:**
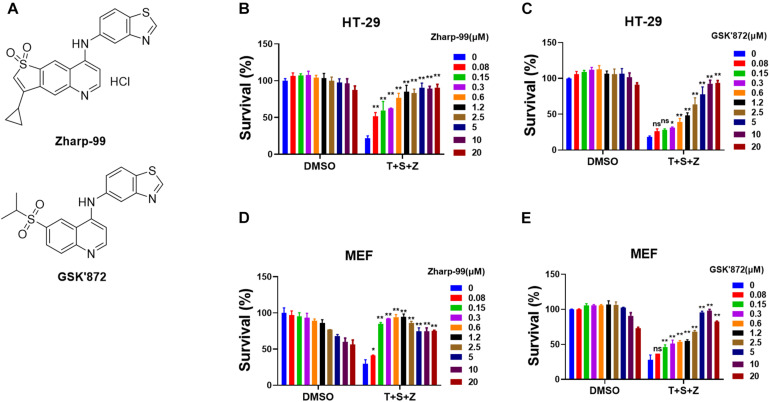
Zharp-99 efficiently blocks TNF-induced necroptosis in both human and mouse cells. **(A)** Chemical structure of Zharp-99 and GSK’872. **(B–E)** The effects of Zharp-99 and GSK’872 on TNF-induced necroptosis were examined in HT-29 cells and MEF cells. **(B,C)** HT-29 cells were pretreated with indicated concentrations of Zharp-99 and GSK’872 for 2 h prior to the treatment with TNF-α (40 ng/ml), Smac mimetic (100 nM) and z-VAD (20 μM) for 48 h. Cell viability was assessed by measuring ATP levels. Data are represented as the mean ± standard deviation of triplicates. **(D,E)** MEF cells were pretreated with indicated concentrations of Zharp-99 and GSK’872 for 2 h followed by the treatment with TNF-α (40 ng/ml), Smac mimetic (100 nM), and z-VAD (20 μM) for 24 h. T, TNF-α; S, Smac mimetic; Z, z-VAD. Data are represented as the mean ± standard deviation of dipartites. *P < 0.05. **P < 0.01.

To discover novel inhibitors of necroptosis, we designed and synthesized a focused library with novel structures based on the GSK’872 scaffold. Human colon cancer HT-29 cells were treated with these compounds for 2 h prior to the treatment of necroptotic stimuli (TNFα, Smac mimetic and z-VAD), which are widely used to trigger TNF-induced necroptosis (He et al., 2009). Zharp-99 turned out to be the most efficient inhibitor of TNF-induced necroptosis in HT-29 cells with higher efficacy compared to the well-known RIPK3 inhibitor GSK’872 (Figures 1A–C). We further examined the effect of Zharp-99 on TNF-induced necroptosis in MEFs. Zharp-99 exhibited efficient inhibition of TNF-induced necroptosis in MEFs at concentrations ranging from 0.15 to 1.2 μM (Figures 1D,E). We also observed obvious toxicity of Zharp-99 at higher concentrations ranging from 2.5 to 20 μM (Figure 1D). Collectively, these results demonstrate that Zharp-99 is an effective inhibitor of TNF-induced necroptosis in human and mouse cells.

### Zharp-99 Inhibits Necroptosis Induced by TLR and HSV-1 Infection

**FIGURE 2:**
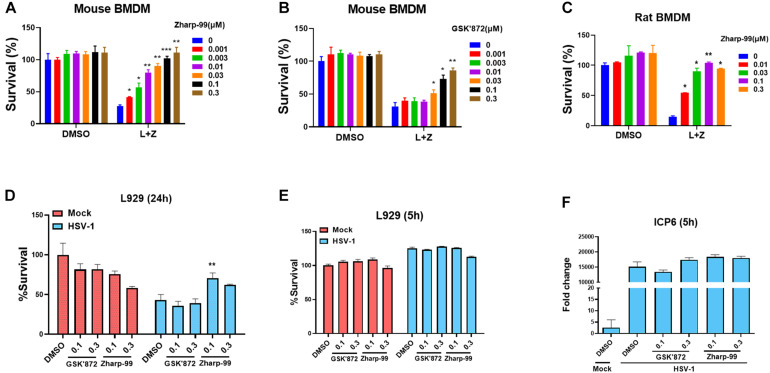
Zharp-99 blocks necroptosis induced by TLR and HSV-1 infection. **(A–C)** Bone marrow-derived macrophages (BMDM) from mouse and rat was pretreated with DMSO, Zharp-99 or GSK’872 for 2 h prior to the treatment of DMSO, LPS (20 ng/ml) plus z-VAD (20 μM) or z-VAD (10 μM) for 24 h. Cell viability was determined by measuring ATP levels. L, LPS; Z, z-VAD. Data are represented as the mean ± standard deviation of dipartites. **(D)** L929 cells were pretreated with DMSO, Zharp-99 or GSK’872 for 2h prior to HSV-1 infection. After 24 h, cell viability was determined by measuring ATP levels. Data are represented as the mean ± standard deviation of triplicates. **(E,F)** L929 cells were pretreated with DMSO, Zharp-99 or GSK’872 for 2 h prior to HSV-1 infection. After 5 h, cell viability was determined by measuring ATP levels and ICP6 mRNA expression was detected using viral genome extracted from cell culture supernatant. *P < 0.05. **P < 0.01. ***P < 0.001.

It is known that necroptosis can be initiated by activation of TLR3 or TLR4 as well as pathogen infection in addition to activation of death receptors (Linkermann and Green, 2014; He and Wang, 2018; Mifflin et al., 2020). We examined the impact of Zharp-99 on TLR4-mediated necroptosis induced by LPS/z-VAD. Zharp-99 potently blocked TLR4-mediated necroptosis in both mouse and rat bone marrow derived macrophages (Figures 2A–C). Infection of herpes simplex virus (HSV)-1 infection can trigger necroptosis in mouse cells (Huang et al., 2015; Wang et al., 2014c). We further evaluated the effect of Zharp-99 on HSV-1-induced necroptosis and found that Zharp-99 significantly inhibited HSV-1 induced necroptosis in L929 cells (Figure 2D). We examined the effect of Zharp-99 on viral genome extracted from cell culture supernatants 5h post HSV-1 infection and found that Zharp-99 did not affect the expression of viral gene ICP6 (Figures 2E,F). These results suggest that Zharp-99 has a common mechanism of blocking conserved necroptosis signaling pathways activated by various stimuli in different species.

### Zharp-99 Blocks Cellular Activation of RIPK3 and MLKL Upon Necroptotic Stimuli

**FIGURE 3:**
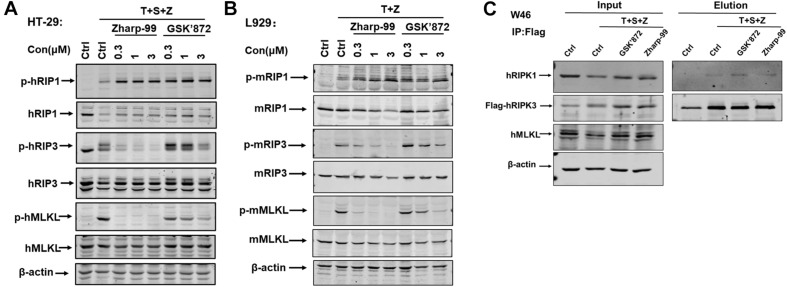
Zharp-99 blocks cellular activation of RIPK3 and MLKL upon necroptotic stimuli. **(A)** HT-29 cells were treated with the indicated compound for 2 h prior to the treatment of TNF-α (40 ng/ml), Smac mimetic (100 nM) and z-VAD (20 μM) for additional 8 h. Cell lysates were harvest and subjected to western-blot analysis for phosphorylation of RIPK1, RIPK3, and MLKL. **(B)** L929 cells were pretreated with DMSO, Zharp-99 or GSK’872 for 2 h prior to TNF-α (40 ng/ml) and z-VAD (20 μM) for additional 4 h. Cell lysates were harvest and subjected to western-blot analysis for phosphorylation of RIPK1, RIPK3, and MLKL. **(C)** HT-29 cells stably expressing RIPK3-shRNA and shRNA resistant scramble Flag-tagged RIPK3 (W46) were pretreated Zharp-99 or GSK’872 for 2 h prior to treatment of TNF-α (40 ng/ml), Smac (100 nM) and z-VAD (20 μM) for 8 h. Cell lysates were prepared for immunoprecipitation using an anti-Flag antibody. The levels of RIPK3 and RIPK1 in the immunocomplex were determined by western blot analysis.

Having established that Zharp-99 is a novel inhibitor of necroptosis, we next investigate the molecular mechanism underlying Zharp-99-mediated necroptosis inhibition. It is well understood that RIPK1, RIPK3, and MLKL are activated during TNF-induced necroptosis, as indicated by their phosphorylation (Sun et al., 2012; Mifflin et al., 2020; Wang et al., 2014a). We examined the effect of Zharp-99 on the phosphorylation of RIPK1, RIPK3, and MLKL upon necroptotic stimuli. Treatment of Zharp-99 abolished phosphorylation of RIPK3 and MLKL in human HT-29 cells, but did not reduce RIPK1 phosphorylation at S166 (Figure 3A). Consistently, Zharp-99 blocked phosphorylation of RIPK3, and MLKL in mouse L929 cells, but not phosphorylation of RIPK1 (Figure 3B). We further examined the effect of Zharp-99 on the formation of RIPK1/RIPK3 complex in HT-29 cells stably expressing Flag-RIPK3. Zharp-99 did not affect the RIPK1/RIPK3 necrosome formation (Figure 3C). Collectively, these results demonstrate that Zharp-99 blocks necroptosis through the suppression of RIPK3 function or signaling upstream of RIPK3 activation.

### Zharp-99 Is an Inhibitor of RIPK3 Kinase Domain

**FIGURE 4:**
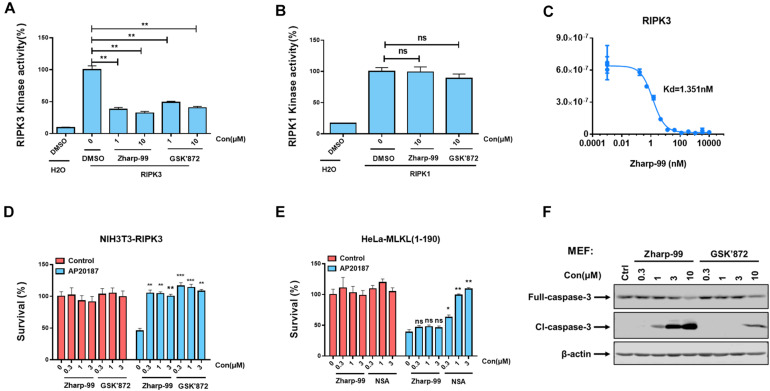
Zharp-99 is a potent inhibitor of RIPK3. **(A,B)** The effect of Zharp-99 on the kinase activities of RIPK3 and RIPK1. In vitro kinase activity assays using recombinant RIPK3 and RIPK1 were performed as described in the section “Materials and Methods.” Data represent mean value ± standard deviation. **(C)** Binding constants (Kds) of Zharp-99 with recombinant human RIPK3 in collaboration with Discover X Corporation. **(D)** Zharp-99 inhibits RIPK3 dimerization-induced cell death in NIH3T3-RIPK3 cells that stably express RIPK3 fused to FKBP F36V mutant. NIH3T3-RIPK3 cells were pretreated with indicated compounds for 2 h and subsequently treated with AP20187 (60 nM) for 24 h. Cell viability was determined by measuring ATP levels. **(E)** Zharp-99 has no effect on MLKL dimerization-induced cell death in HeLa-MLKL (1–190) cells that stably express MLKL (1–190aa) fused to DmrB. HeLa-MLKL (1–190) cells were induced by doxycycline (1 μg/ml) for 24 h. Cells were pretreated with indicated compounds for 2 h and subsequently treated with AP20187 (60nM) for 24h. Cell viability was determined by measuring ATP levels. Data are represented as the mean ± standard deviation of triplicates. *P < 0.05. **P < 0.01. ***P < 0.001. **(F)** MEFs were treated as indicated for 3 h. Cell lysates were harvest and subjected to western-blot analysis for full length and cleaved caspase 3.

Having shown that Zharp-99 can block activation of RIPK3 and MLKL during TNF-induced necroptosis, we further asked whether Zharp-99 could directly target RIPK3 by performing *in vitro* kinase assay. Zharp-99 inhibited the kinase activity of human RIPK3 *in vitro* with higher inhibitory activity compared to GSK’872 (Figure 4A). In contrast, Zharp-99 did not affect RIPK1 kinase activity even at 10 μM, displaying a similar effect as GSK’872 (Figure 4B). Moreover, Zharp-99 exhibited efficient binding to human recombinant RIPK3 with Kd of 1.35 nM (Figure 4C). These results indicate that Zharp-99 is an inhibitor of RIPK3 by targeting the kinase activity.

Previous studies have shown that enforced dimerization/polymerization of RIPK3 or MLKL triggers necroptosis bypassing the upstream signals (Chen et al., 2014; Orozco et al., 2014). We further evaluated the effect of Zharp-99 on necroptosis induced by RIPK3 dimerization or MLKL polymerization. Consistent with previous observation, NIH3T3 cells expressing mouse RIPK3 fused to mutant FK506-binding protein (FKBP) were committed to necroptosis upon the treatment of the dimerizer AP20187 (Figure 4D). This RIPK3 dimerization-induced necroptosis was efficiently blocked by Zharp-99 (Figure 4D). It has been reported that HeLa cells expressing MLKL (1–190aa) fused to DmrB could undergo MLKL polymerization-induced necroptosis upon AP20187 treatment (Liu et al., 2017; Figure 4E). Treatment of Zharp-99 did not affect this polymerized MLKL-induced necroptosis, while the cell death phenotype was blocked by MLKL inhibitor necrosulfonamide (NSA), suggesting that Zharp-99 does not affect MLKL function or signaling downstream of MLKL. Taken together, these results demonstrate that Zharp-99 inhibits necroptosis via the blockage of RIPK3 kinase activity. It has been demonstrated that GSK’872 is able to induce on-target apoptosis (Mandal et al., 2014). Consistently, Zharp-99 induced activation of caspase-3 and cell death in a dose-dependent manner especially in mouse cells (Figures 1D,4F). Compared to GSK’872, Zharp-99 was more potent in inducing apoptosis in MEFs (Figures 1D,E, 4F).

### Zharp-99 Effectively Alleviates TNF-Induced SIRS

**FIGURE 5:**
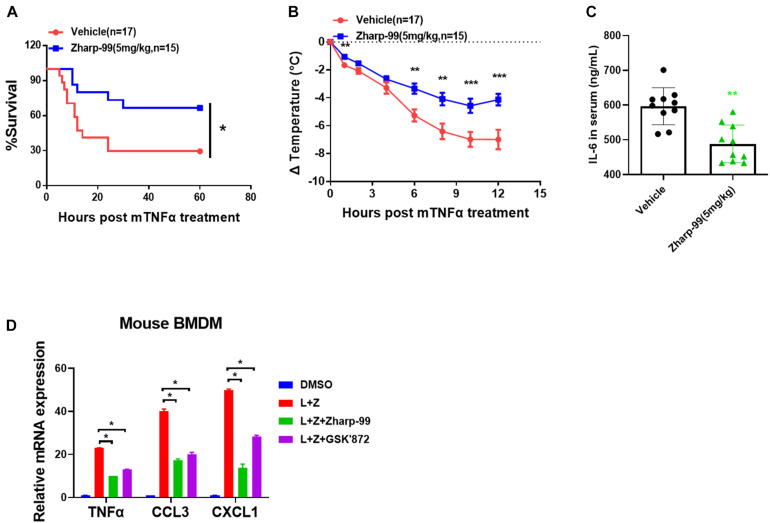
Zharp-99 effectively alleviates TNF-induced systemic inflammatory response syndrome. C57BL/6 mice were injected with vehicle or Zharp-99 (5 mg/kg) via intraperitoneal injection for around 15 min, followed by the tail intravenous injection of mouse TNF-α (6.5 μg/mouse). **(A,B)** The survival rate **(A)** and body temperature loss **(B)** were monitored. The body temperature changes (means ± SEM) for each group are shown. *P*-values were determined using the ONE-way ANOVA and multi-comparison test for statistics analysis. **(C)** After 4 h, serum was collected for mouse IL6 level analysis by ELISA. Data are represented as the mean ± standard deviation. **(D)** Mouse BMDM were pretreated with DMSO, Zharp-99 or GSK’872 for 2 h prior to the treatment of DMSO or LPS (20 ng/ml) plus z-VAD (20 μM) as indicated. After 7 h, cell were harvested for analysis of mRNA expression of TNFα, CCL3, CXCL1 or IL-6 by Q-PCR. *P < 0.05. **P < 0.01. ***P < 0.001.

Based on the strong biochemical and cellular anti-necroptosis activity of Zharp-99, we sought to test the therapeutic potential of Zharp-99 in the mouse model of necroptosis-associated diseases. Necroptosis is associated with various pathological conditions such as TNF-induced SIRS (Berger et al., 2014; Duprez et al., 2011). We evaluated the protective effect of Zharp-99 in TNF-induced SIRS in vivo. Female C57BL/6 mice were treated with vehicle or Zharp-99 for 15 min, followed by the intravenous injection of mouse TNFα at 6.5 μg/mouse. Treatment with 5 mg/kg Zharp-99 significantly protected mice against TNFα-induced lethal shock (Figure 5A). Zharp-99 treatment also ameliorated TNFα-induced temperature loss in mice (Figure 5B). Moreover, Zharp-99 reduced TNFα-induced production of IL-6 in the serum (Figure 5C). Collectively, these results demonstrate that RIPK3 inhibition by Zharp-99 provides effective protection against TNF-induced SIRS. The kinase activity of RIPK3 was shown to be required for RIPK3-mediated expression of inflammatory cytokines in mouse BMDM treated with LPS plus z-VAD (Najjar et al., 2016). We found that Zharp-99 could inhibit LPS/z-VAD-induced expression of inflammatory cytokines including TNFα, CCL3, and CXCL1 in mouse BMDM (Figure 5D). Taken together, these results suggest that Zharp-99 inhibits RIPK3-mediated cytokine production both *in vitro* and *in vivo*.

### Zharp-99 Displays Favorable *in vitro* Safety Profiles and *in vivo* Pharmacokinetic Parameters

**FIGURE 6:**
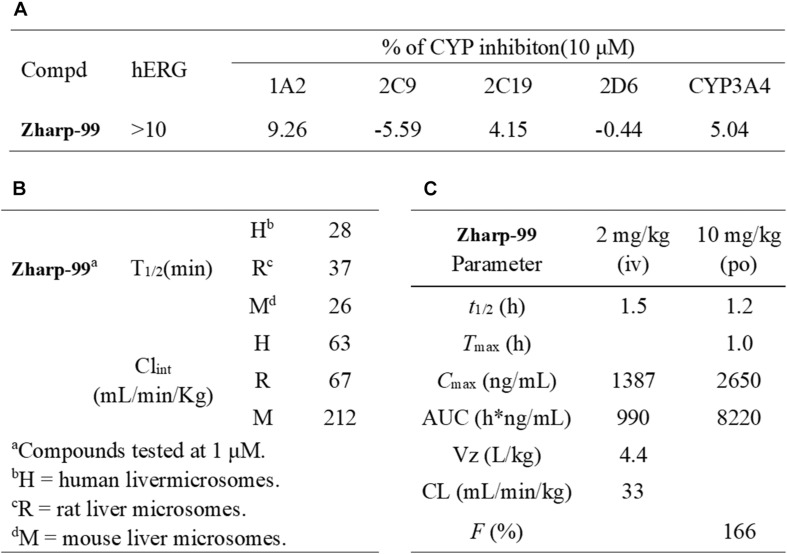
Zharp-99 displays favorable in vitro safety profiles and in vivo pharmacokinetic parameters. **(A)** CYP and hERG inhibition of Zharp-99. **(B)** Metabolic stability of Zharp-99 in human, rat, and mouse liver microsomes. **(C)** Pharmacokinetic parameters of Zharp-99 determined in male ICR mouse.

Encouraged by the *in vitro* and *in vivo* efficacy data, we sought to evaluate the preliminary drugability profile of Zharp-99. Zharp-99 did not inhibit major human cytochrome P450 isozymes (CYP3A4, 2D6, 1A2, 2C9, 2C19) at 10 μM concentration, suggesting low liability for potential drug/drug interactions. Moreover, Zharp-99 exhibited low inhibition (IC50 > 10 μM) of hERG (standard patch clamp), indicating minimal cardiotoxicity associated with blockade of this key potassium channel (Figure 6A). When evaluated for its in vitro metabolic stability in mouse, rat and human liver microsomes, Zharp-99 demonstrated moderate intrinsic clearance across rodent and human species, leading to half-lives ranging from 26 min (MLM) to 37 min (RLM, Figure 6B). This data was recapitulated in the standard in vivo mouse pharmacokinetic study. When dosed orally at 10 mg/kg in mice, Zharp-99 was quickly absorbed with a T_max_ of 1 h and C_max_ of 2650 ng/mL. Zharp-99 exhibited a moderate clearance (33 mL/min/kg) and volume of distribution (4.4 L/kg) with a short half-life (1.5 h). The exposure was 8220 h ng/mL, leading to an estimated oral bioavailability over 100% (Figure 6C).

## Discussion

Necroptosis plays a pivotal role in the pathogenesis of diseases including inflammatory diseases, neurodegenerative diseases, ischemia-reperfusion induced tissue injury and cancer (Linkermann and Green, 2014; He and Wang, 2018; Mifflin et al., 2020). Although RIPK1 is considered as a promising therapeutic targets, emerging evidence suggests that RIPK1-independent necroptosis proceeds when RIPK3 is activated by other RHIM-containing proteins (Mifflin et al., 2020). In the present study, we discovered Zharp-99 as a novel necroptosis inhibitor that directly blocks the kinase activity of RIPK3 and efficiently inhibits necroptosis *in vitro* and *in vivo*.

The kinase activity of RIPK3 is essential for necroptosis (Cho et al., 2009; He et al., 2009; Zhang et al., 2009). Therefore, inhibition of RIPK3 kinase activity is an attractive strategy for interfering with necroptosis and necroptosis-related injury. Our study has demonstrated that Zharp-99 is a potent inhibitor of RIPK3 kinase activity with Kd of 1.35nM. Zharp-99 exhibits higher efficacy in the inhibition of necroptosis and RIPK3 kinase activity compared to GSK872, a well characterized RIPK3 inhibitor (Kaiser et al., 2013; Mandal et al., 2014). It has been noted that mice expressing catalytically inactive RIPK3 D161N leads to caspase8-dependent embryonic lethality (Newton et al., 2014). This phenomenon raises concerns regarding the possible toxic effect in the whole animal when RIPK3 kinase activity is impaired. Unlike RIPK3 D161N knock-in mice, RIPK3 kinase inactive mice which carry a RIPK3 K51A knock-in mutation develop normally and are fertile (Mandal et al., 2014), providing the evidence that inhibition of RIPK3 kinase activity can be tolerated in mice depending on the context. Moreover, the kinase-inactive mutant forms of RIPK3 K51A, D143N, and D161G do not induce apoptosis (Mandal et al., 2014). These findings suggest that the kinase activity of RIP3 kinase is not vital for cell survival. It has been reported that several RIPK3 kinase inhibitors trigger apoptosis by exposing its RHIM domain, therefore facilitating RIPK1 recruitment and activation of caspase-8 for apoptosis (Mandal et al., 2014). Although Zharp-99 does not cause obvious cell death at the tested concentrations when it completely blocks necroptosis, it induces apoptosis at higher concentrations.

Importantly, Zharp-99 provides strong protection against TNF-induced lethal shock and inflammatory responses in the mouse model. This result supports an essential role of RIPK3 kinase activity in the pathogenesis of TNF-induced systemic inflammatory injury. Our work highlights the potential of Zharp-99 for the development of novel anti-inflammatory therapies based on RIPK3 inhibition. It is worth noting that RIPK3 has been shown to regulate inflammatory signaling pathways via a necroptosis-independent mechanism (Wang et al., 2014b; Lawlor et al., 2015; Yatim et al., 2015; Moriwaki and Chan, 2016; Najjar et al., 2016; Daniels et al., 2017). Therefore, further investigation in various mouse models of human disease using RIPK3 kinase-dead mice and RIPK3 inhibitors will provide crucial insights for developing valuable therapies targeting RIPK3.

## Data Availability Statement

The original contributions presented in the study are included in the article/Supplementary Materials, further inquiries can be directed to the corresponding authors.

## Ethics Statement

The animal study was reviewed and approved by The Institutional Animal Care and Use Committee at Suzhou Institute of Systems Medicine.

## Author Contributions

SH and XZ designed the study and revised the manuscript. KX and FZ performed the molecular biology and chemistry studies and animal model, analyzed the data, and drafted the manuscript. CY XY, CZ, YJ, WG, JW, and YD performed the cell culture, biochemistry, kinase assay, and HSV infection. SW, YL, and HM performed the chemical synthesis. TY and WZ performed the cell culture and viability assay. All authors contributed to the article and approved the submitted version.

## Conflict of Interest

XZ and SH are co-founders, consultants and shareholders of Accro Bioscience Inc., which supports research in their labs. The remaining authors declare that the research was conducted in the absence of any commercial or financial relationships that could be construed as a potential conflict of interest.

## References

[B1] Berger S. B. Kasparcova V. Hoffman S. Swift B. Dare L. Schaeffer M. (2014). Cutting Edge: RIP1 kinase activity is dispensable for normal development but is a key regulator of inflammation in SHARPIN-deficient mice. *J. Immunol.* 192 5476–5480. 10.4049/jimmunol.1400499 24821972 PMC4048763

[B2] Cai Z. Jitkaew S. Zhao J. Chiang H. C. Choksi S. Liu J. (2014). Plasma membrane translocation of trimerized MLKL protein is required for TNF-induced necroptosis. *Nat. Cell Biol.* 16 55–65. 10.1038/ncb2883 24316671 PMC8369836

[B3] Chen X. Li W. Ren J. Huang D. He W. T. Song Y. (2014). Translocation of mixed lineage kinase domain-like protein to plasma membrane leads to necrotic cell death. *Cell Res.* 24 105–121. 10.1038/cr.2013.171 24366341 PMC3879712

[B4] Cho Y. S. Challa S. Moquin D. Genga R. Ray T. D. Guildford M. (2009). Phosphorylation-driven assembly of the RIP1-RIP3 complex regulates programmed necrosis and virus-induced inflammation. *Cell* 137 1112–1123. 10.1016/j.cell.2009.05.037 19524513 PMC2727676

[B5] Daniels B. P. Snyder A. G. Olsen T. M. Orozco S. Oguin T. H. III Tait S. W. G. (2017). RIPK3 restricts viral pathogenesis via cell death-independent neuroinflammation. *Cell* 169:e11. 10.1016/j.cell.2017.03.011 28366204 PMC5405738

[B6] Dong Y. Li K. Xu Z. Ma H. Zheng J. Hu Z. (2015). Exploration of the linkage elements of porcupine antagonists led to potent Wnt signaling pathway inhibitors. *Bioorg. Med. Chem.* 23 6855–6868. 10.1016/j.bmc.2015.09.048 26455655

[B7] Duprez L. Takahashi N. Van Hauwermeiren F. Vandendriessche B. Goossens V. Vanden Berghe T. (2011). RIP kinase-dependent necrosis drives lethal systemic inflammatory response syndrome. *Immunity.* 35 908–918. 10.1016/j.immuni.2011.09.020 22195746

[B8] Fauster A. Rebsamen M. Huber K. V. Bigenzahn J. W. Stukalov A. Lardeau C. H. (2015). A cellular screen identifies ponatinib and pazopanib as inhibitors of necroptosis. *Cell Death Dis.* 6:e1767. 10.1038/cddis.2015.130 25996294 PMC4669708

[B9] Hart A. C. Abell L. Guo J. Mertzman M. E. Padmanabha R. Macor J. E. (2020). Identification of RIPK3 type II inhibitors using high-throughput mechanistic studies in hit triage. *ACS Med. Chem. Lett.* 11 266–271. 10.1021/acsmedchemlett.9b00065 32184955 PMC7073880

[B10] He S. Wang X. (2018). RIP kinases as modulators of inflammation and immunity. *Nat. Immunol.* 19 912–922. 10.1038/s41590-018-0188-x 30131615

[B11] He S. Liang Y. Shao F. Wang X. (2011). Toll-like receptors activate programmed necrosis in macrophages through a receptor-interacting kinase-3-mediated pathway. *Proc. Natl. Acad. Sci. U S A.* 108 20054–20059. 10.1073/pnas.1116302108 22123964 PMC3250173

[B12] He S. Wang L. Miao L. Wang T. Du F. Zhao L. (2009). Receptor interacting protein kinase-3 determines cellular necrotic response to TNF-alpha. *Cell* 137 1100–1111. 10.1016/j.cell.2009.05.021 19524512

[B13] Holler N. Zaru R. Micheau O. Thome M. Attinger A. Valitutti S. (2000). Fas triggers an alternative, caspase-8-independent cell death pathway using the kinase RIP as effector molecule. *Nat. Immunol.* 1 489–495. 10.1038/82732 11101870

[B14] Huang Z. Wu S. Q. Liang Y. Zhou X. Chen W. Li L. (2015). RIP1/RIP3 binding to HSV-1 ICP6 initiates necroptosis to restrict virus propagation in mice. *Cell Host. Microbe.* 17 229–242. 10.1016/j.chom.2015.01.002 25674982

[B15] Kaiser W. J. Sridharan H. Huang C. Mandal P. Upton J. W. Gough P. J. (2013). Toll-like receptor 3-mediated necrosis via TRIF. RIP3, and MLKL. *J. Biol. Chem.* 288 31268–31279. 10.1074/jbc.M113.462341 24019532 PMC3829437

[B16] Lawlor K. E. Khan N. Mildenhall A. Gerlic M. Croker B. A. D’Cruz A. A. (2015). RIPK3 promotes cell death and NLRP3 inflammasome activation in the absence of MLKL. *Nat. Commun.* 6:6282. 10.1038/ncomms7282 25693118 PMC4346630

[B17] Li J. X. Feng J. M. Wang Y. Li X. H. Chen X. X. Su Y. (2014). The B-Raf(V600E) inhibitor dabrafenib selectively inhibits RIP3 and alleviates acetaminophen-induced liver injury. *Cell Death Dis.* 5:e1278. 10.1038/cddis.2014.241 24901049 PMC4611716

[B18] Linkermann A. Green D. R. (2014). Necroptosis. *N. Engl. J. Med.* 370 455–465. 10.1056/NEJMra1310050 24476434 PMC4035222

[B19] Liu S. Liu H. Johnston A. Hanna-Addams S. Reynoso E. Xiang Y. (2017). MLKL forms disulfide bond-dependent amyloid-like polymers to induce necroptosis. *Proc. Natl. Acad. Sci. U S A.* 114 E7450–E7459. 10.1073/pnas.1707531114 28827318 PMC5594682

[B20] Lu W. Liu Y. Ma H. Zheng J. Tian S. Sun Z. (2017). Design. synthesis, and structure-activity relationship of tetrahydropyrido[4,3-d]pyrimidine derivatives as potent smoothened antagonists with in vivo activity. *ACS Chem. Neurosci.* 8 1980–1994. 10.1021/acschemneuro.7b00153 28618224

[B21] Mandal P. Berger S. B. Pillay S. Moriwaki K. Huang C. Guo H. (2014). RIP3 induces apoptosis independent of pronecrotic kinase activity. *Mol. Cell* 56 481–495. 10.1016/j.molcel.2014.10.021 25459880 PMC4512186

[B22] Mifflin L. Ofengeim D. Yuan J. (2020). Receptor-interacting protein kinase 1 (RIPK1) as a therapeutic target. *Nat. Rev. Drug. Discov.* 19 553–571. 10.1038/s41573-020-0071-y 32669658 PMC7362612

[B23] Moriwaki K. Chan F. K. (2016). Necroptosis-independent signaling by the RIP kinases in inflammation. *Cell Mol. Life Sci.* 73 2325–2334. 10.1007/s00018-016-2203-4 27048814 PMC4889460

[B24] Murphy J. M. Czabotar P. E. Hildebrand J. M. Lucet I. S. Zhang J. G. Alvarez-Diaz S. (2013). The pseudokinase MLKL mediates necroptosis via a molecular switch mechanism. *Immunity* 39 443–453. 10.1016/j.immuni.2013.06.018 24012422

[B25] Najjar M. Saleh D. Zelic M. Nogusa S. Shah S. Tai A. (2016). RIPK1 and RIPK3 kinases promote cell-death-independent inflammation by toll-like receptor 4. *Immunity* 45 46–59. 10.1016/j.immuni.2016.06.007 27396959 PMC4956514

[B26] Newton K. Dugger D. L. Wickliffe K. E. Kapoor N. de Almagro M. C. Vucic D. (2014). Activity of protein kinase RIPK3 determines whether cells die by necroptosis or apoptosis. *Science* 343 1357–1360. 10.1126/science.1249361 24557836

[B27] Orozco S. Yatim N. Werner M. R. Tran H. Gunja S. Y. Tait S. W. (2014). RIPK1 both positively and negatively regulates RIPK3 oligomerization and necroptosis. *Cell Death Differ.* 21 1511–1521. 10.1038/cdd.2014.76 24902904 PMC4158689

[B28] Park H. H. Park S. Y. Mah S. Park J. H. Hong S. S. Hong S. (2018). HS-1371, a novel kinase inhibitor of RIP3-mediated necroptosis. *Exp. Mol. Med.* 50:125. 10.1038/s12276-018-0152-8 30237400 PMC6148246

[B29] Samir P. Malireddi R. K. S. Kanneganti T. D. (2020). The PANoptosome: a deadly protein complex driving pyroptosis. apoptosis, and necroptosis (PANoptosis). *Front. Cell Infect. Microbiol.* 10:238. 10.3389/fcimb.2020.00238 32582562 PMC7283380

[B30] Sun L. Wang H. Wang Z. He S. Chen S. Liao D. (2012). Mixed lineage kinase domain-like protein mediates necrosis signaling downstream of RIP3 kinase. *Cell* 148 213–227. 10.1016/j.cell.2011.11.031 22265413

[B31] Upton J. W. Kaiser W. J. Mocarski E. S. (2012). DAI/ZBP1/DLM-1 complexes with RIP3 to mediate virus-induced programmed necrosis that is targeted by murine cytomegalovirus vIRA. *Cell Host. Microbe.* 11 290–297. 10.1016/j.chom.2012.01.016 22423968 PMC3531981

[B32] Wang H. Sun L. Su L. Rizo J. Liu L. Wang L. F. (2014a). Mixed lineage kinase domain-like protein MLKL causes necrotic membrane disruption upon phosphorylation by RIP3. *Mol. Cell* 54 133–146. 10.1016/j.molcel.2014.03.003 24703947

[B33] Wang L. Du F. Wang X. (2008). TNF-alpha induces two distinct caspase-8 activation pathways. *Cell* 133 693–703. 10.1016/j.cell.2008.03.036 18485876

[B34] Wang X. Jiang W. Yan Y. Gong T. Han J. Tian Z. (2014b). RNA viruses promote activation of the NLRP3 inflammasome through a RIP1-RIP3-DRP1 signaling pathway. *Nat. Immunol.* 15 1126–1133. 10.1038/ni.3015 25326752

[B35] Wang X. Li Y. Liu S. Yu X. Li L. Shi C. (2014c). Direct activation of RIP3/MLKL-dependent necrosis by herpes simplex virus 1 (HSV-1) protein ICP6 triggers host antiviral defense. *Proc. Natl. Acad. Sci. U S A.* 111 15438–15443. 10.1073/pnas.1412767111 25316792 PMC4217423

[B36] Yatim N. Jusforgues-Saklani H. Orozco S. Schulz O. Barreira R. (2015). da Silva. C Reis e Sousa, et al. RIPK1 and NF-kappaB signaling in dying cells determines cross-priming of CD8(+) T cells. *Science* 350 328–334. 10.1126/science.aad0395 26405229 PMC4651449

[B37] Zhang D. W. Shao J. Lin J. Zhang N. Lu B. J. Lin S. C. (2009). RIP3, an energy metabolism regulator that switches TNF-induced cell death from apoptosis to necrosis. *Science* 325 332–336. 10.1126/science.1172308 19498109

[B38] Zhang H. Xu L. Qin X. Chen X. Cong H. Hu L. (2019). N-(7-Cyano-6-(4-fluoro-3-(2-(3-(trifluoromethyl)phenyl)acetamido)phenoxy)benzo[d] thiazol-2-yl)cyclopropanecarboxamide (TAK-632) analogues as novel necroptosis inhibitors by targeting receptor-interacting protein kinase 3 (RIPK3): synthesis. structure-activity relationships, and in vivo efficacy. *J. Med. Chem.* 62 6665–6681. 10.1021/acs.jmedchem.9b00611 31095385

[B39] Zhao J. Jitkaew S. Cai Z. Choksi S. Li Q. Luo J. (2012). Mixed lineage kinase domain-like is a key receptor interacting protein 3 downstream component of TNF-induced necrosis. *Proc. Natl. Acad. Sci. U S A.* 109 5322–5327. 10.1073/pnas.1200012109 22421439 PMC3325682

