# Supplementary material for: Discovery of a Potent RIPK3 Inhibitor for the Amelioration of Necroptosis-Associated Inflammatory Injury
Source: Front Cell Dev Biol. 2020 Dec 8;8:606119. doi: 10.3389/fcell.2020.606119 (PMC7753040; doi:10.3389/fcell.2020.606119)
Supplement: Supplementary file 1 [file Data_Sheet_1.PDF]

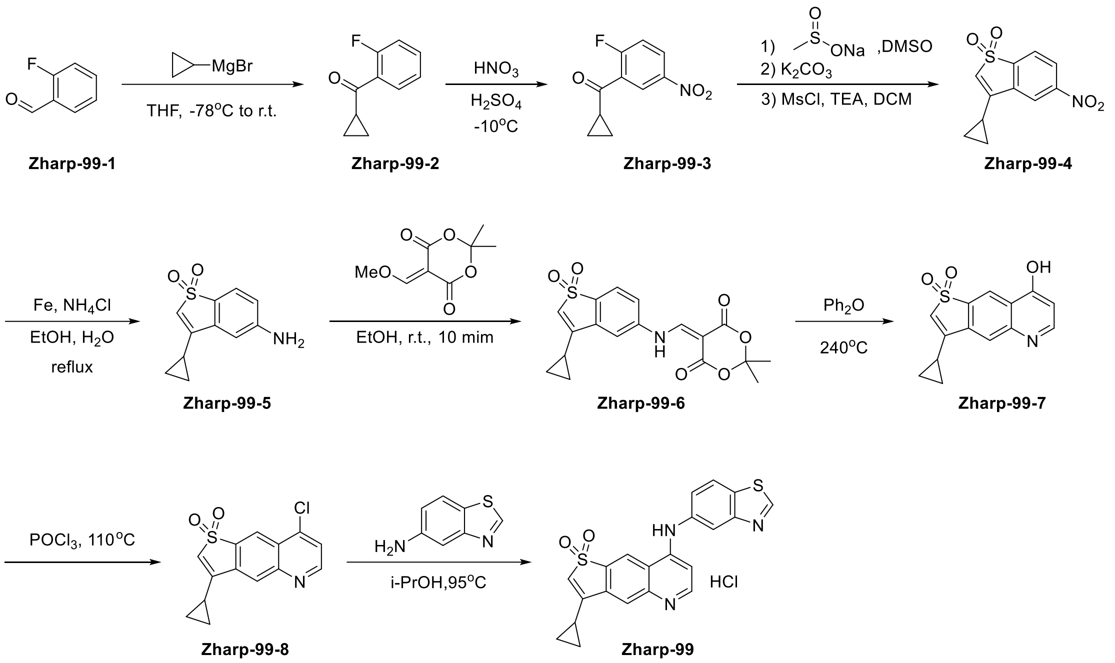

Supplementary Figure 7a. Synthetic scheme for **Zharp-99**.

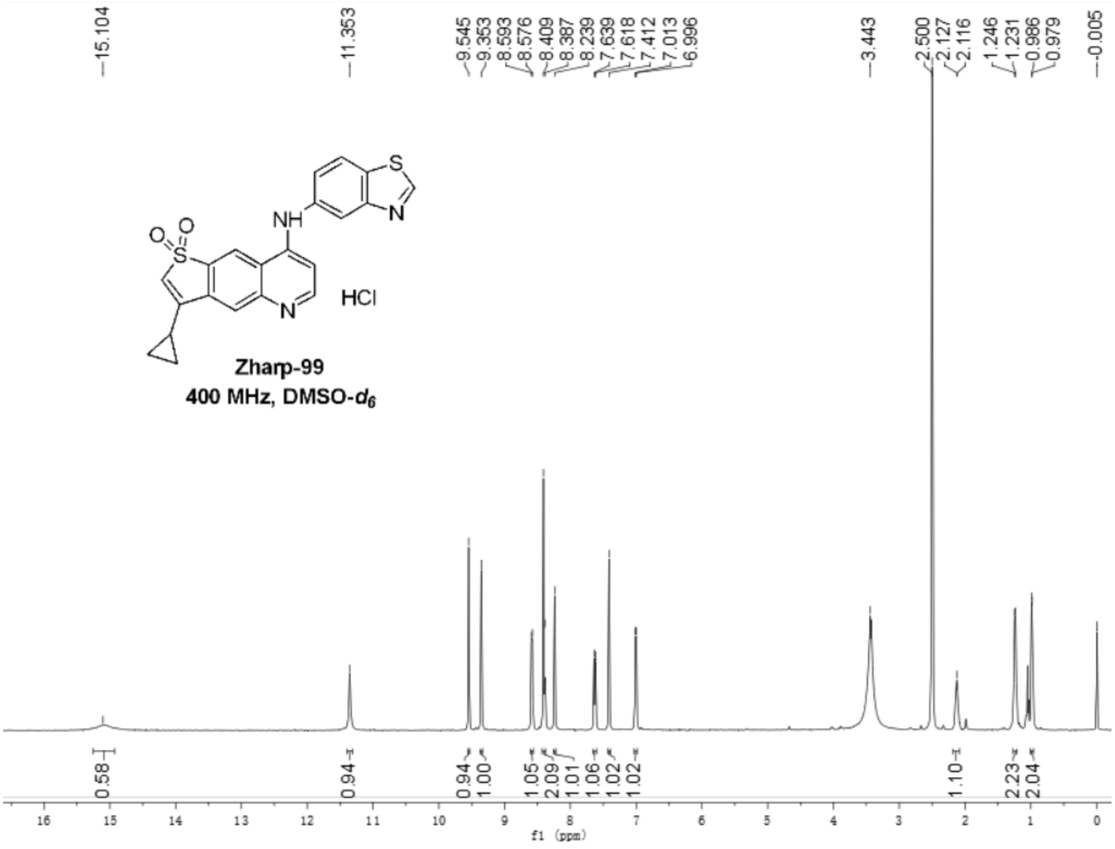

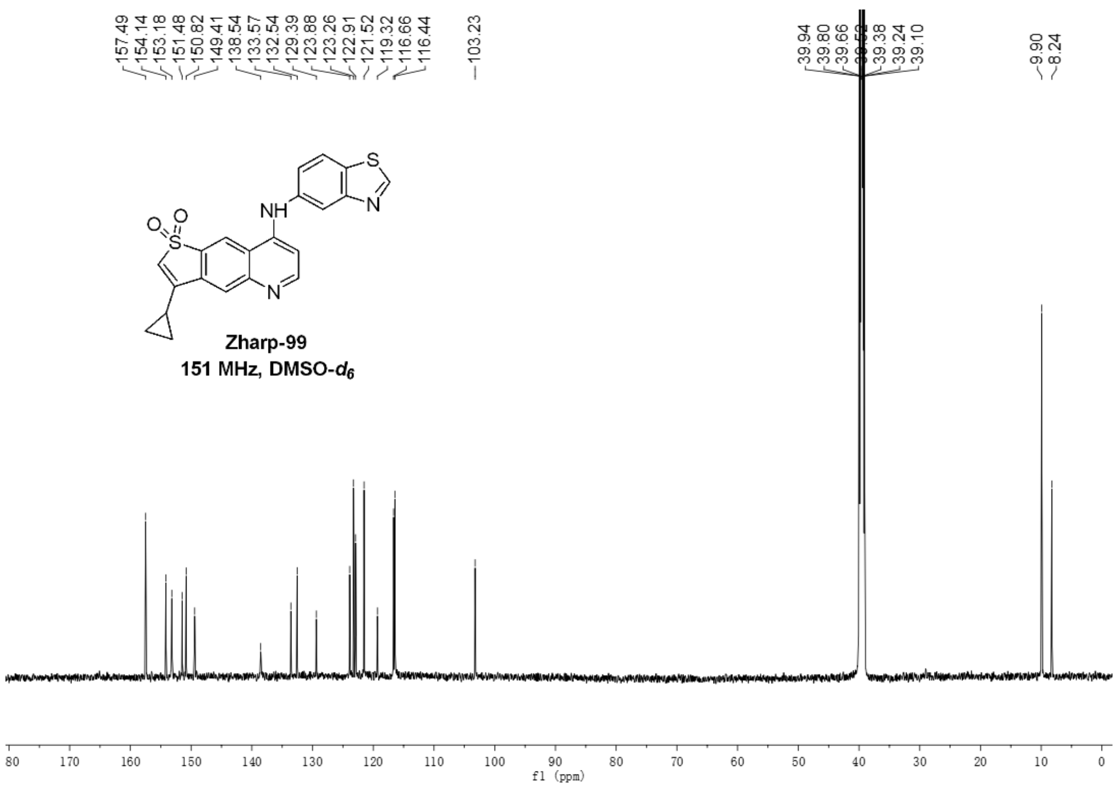

Supplementary Figure 7b. ^{1}H (HCl salt) and ^{13}C NMR (free base) spectra for **Zharp-99**

Supplementary Methods

Synthetic procedure

General reaction progress was monitored by analytical thin layer chromatography performed on silica gel HSGF254 pre-coated plates. Organic solutions were dried over anhydrous Na_2SO_4, and the solvents were removed under reduced pressure. Final compounds were purified with silica gel 100-200 mesh for column chromatography. ^{1}H NMR and ^{13}C NMR were obtained on 400 MHz (Varian) or 600 MHz (Varian) spectrometers. Chemical shifts were given in ppm using tetramethylsilane as internal standard. Data for ^{1}H NMR are reported as follows: chemical shift, multiplicity (s = singlet, d = doublet, t = triplet, q = quartet, m = multiplet, br = broad), coupling constants and integration. Mass spectra were obtained using an Agilent 1100 LC/MSD Trap SL version Mass Spectrometer. HRMS analysis was recorded on an Agilent 6540 UHD Accurate-Mass QTOF LC/MS. HPLC method: Waters Acquity UPLC, BEH C18 2.1 mm × 50 mm, 1.7 μm particles. Mobile phase A: 5 mM aqueous formic acid. Mobile phase B: MeOH. Temperature: 24 °C. Gradient: 5−40% B over 1 min, 40-70% B over 1 min, 70-95% B over 4 min, then a 1 min hold at 95% B. Flow: 1.2 mL/min. Detection: UV at 254 nm. Melting points were determined by a SMP10 melting point apparatus.

Zharp-99-2: cyclopropyl(2-fluorophenyl)methanone

To a solution of **Zharp-99-1** (3.7 g, 30 mmol) in tetrahydrofuran (30 mL) at -78 °C was

added dropwise cyclopropylmagnesium bromide (1 N, 30 mL, 30 mmol). The mixture was stirred at room temperature overnight. Sat. aq. NH_4Cl (30 mL) was added to quench the reaction. The solution was extracted with ethyl acetate (50 mL * 2). The combined organic layer was dried over Na_2SO_4 and concentrated. The residue was purified by silica gel column chromatography (petroleum ether/ethyl acetate = 50 / 1) to give the desired product (4.3 g, 87%) as a yellow oil. ^{1}H NMR (400 MHz, $CDCl_3$) δ 7.80-7.69 (m, 1H), 7.54-7.45 (m, 1H), 7.23-7.19 (m, 1H), 7.19-7.14 (m, 1H), 2.73-2.59 (m, 1H), 1.34-1.21 (m, 2H), 1.16-1.00 (m, 2H). LC-MS (m/z): 164.9 $[M+H]^+$.

Zharp-99-3: cyclopropyl(2-fluoro-5-nitrophenyl)methanone

To a stirred slurry of conc. H_2S0_4 (98%, 15 mL) at -10 °C were added dropwise **Zharp-99-2** (4.3 g, 26 mmol) and fuming nitric acid (1.7 mL, 26 mmol). The slurry was stirred for 30 min at -10 °C. The mixture was slowly poured onto ice water (30 mL). The solution was extracted with dichloromethane (30 mL * 2). The combined organic layer was dried over Na_2SO_4 and concentrated. The residue was purified by silica gel column chromatography (petroleum ether/ethyl acetate = 50 / 1) to give the desired product (3.7 g, 68%) as a colorless oil. ^{1}H NMR (400 MHz, $CDCl_3$) δ 8.70-8.62 (m, 1H), 8.44-8.33 (m, 1H), 7.40-7.29 (m, 1H), 7.19-7.14 (m, 1H), 2.71-2.56 (m, 1H), 1.41-1.31 (m, 2H), 1.21-1.10 (m, 2H).

Zharp-99-4: 3-cyclopropyl-5-nitrobenzo[*b*]thiophene 1,1-dioxide

To a solution of **Zharp-99-3** (3.7 g, 17.7 mmol) in *N*, *N*-dimethylformamide (10 mL) was added sodium methanesulfinate (1.8 g, 17.7 mmol). The mixture was stirred at room temperature for 1 h. K_2CO_3 (49 g, 35.4 mmol) was added and the mixture was stirred at 70 °C overnight. Water (50 mL) was added and the solution was extracted with ethyl acetate (100 mL * 2). The combined organic layer was dried over Na_2SO_4 and concentrated. The residue was dissolved in dichloromethane. Triethylamine (7.2 g, 70.8 mmol) and methanesulfonyl chloride (4.0 g, 35.4 mmol) were added. The mixture was stirred at room temperature overnight. The organic layer was removed and the residue was purified by silica gel column chromatography (petroleum ether/ethyl acetate = 2 / 1) to give the desired product (2.7 g, 62%) as a yellow solid. ^{1}H NMR (400 MHz, $CDCl_3$) δ 8.53-8.42 (m, 2H), 7.89 (d, J = 8.8 Hz, 1H), 6.33 (d, J = 1.2 Hz, 1H), 1.96-1.88 (m, 1H), 1.29-1.25 (m, 2H), 0.92-0.85 (m, 2H).

Zharp-99-5: 5-amino-3-cyclopropylbenzo[*b*]thiophene 1,1-dioxide

To a solution of **Zharp-99-4** (2.7 g, 11 mmol) in ethanol (30 mL) and water (8 mL) was added iron powder (3.1mg, 55 mmol) and ammonium chloride (2.9 g, 55 mmol). The mixture was stirred at 85°C for 2 hours. The solution was filtered via diatomite. The filtrate was extracted with dichloromethane (100 mL * 3). The combined organic layer was dried over Na_2SO_4 and concentrated. The residue was purified by silica gel column chromatography (petroleum ether/ethyl acetate = 1 / 1) to give the desired product (1.8 g, 74%) as a yellow solid. ^{1}H NMR (400 MHz, $CDCl_3$) δ7.44 (d, J = 8.0 Hz, 1H), 6.81 (s, 1H),6.66 (d, J = 7.6 Hz, 1H), 6.11 (s, 1H), 4.21 (s, 2H),1.78-1.74 (m, 1H), 1.26-1.23 (m, 2H), 087-0.84 (m, 2H). LCMS (ESI/APCI) m/z: 238.9 $[M+NH_4]^+$.

Zharp-99-6: 5-(((3-cyclopropyl-1,1-dioxidobenzo[b]thiophen-5-yl)amino)methylene)-2,2-dimethyl-1,3-dioxane-4,6-dione

To a solution of **Zharp-99-5** (1.8 g, 8.1 mmol) in ethanol (30 mL) was added 5-(methoxymethylene)-2,2-dimethyl-1,3-dioxane-4,6-dione (3.0 g, 16.2 mmol) slowly. The mixture was stirred at room temperature for 30 minutes. The product was precipitated from the solution. The product was collected via filtration, washed with ethanol (10 mL) and dried in vacuum to give the desired product (2.8 g, 92%) as a yellow solid. ^{1}H NMR (400 MHz, $CDCl_3$) δ 11.41 (d, *J* = 13.6 Hz, 1H), 8.70 (d, *J* = 14.0 Hz, 1H), 7.76 (d, *J* = 8.4 Hz, 1H), 7.45 (s, 1H), 7.37 (d, *J* = 8.4 Hz, 1H), 6.27 (s, 1H), 2.09-1.94 (m, 1H), 1.78 (s, 6H), 1.24-1.20 (m, 2H), 0.87-0.82 (m, 2H).

Zharp-99-7: 3-cyclopropyl-8-hydroxythieno[2,3-*g*]quinoline 1,1-dioxide

The diphenyl ether (20 mL) was added to a round-bottomed flask and the solvent was heated to 240°C for 5 minutes. Intermediate **Zharp-99-6** (500 mg, 1.4 mmol) was added dropwise to the solution. The mixture was stirred for 5 minutes. After cooling to room temperature, the solid was precipitated from the solution. The solid was collected via filtration, washed with ether (20 mL) and dried in vacuum to give the desired product **Zharp-99-7** (200 mg, 52%) as a grey solid. ^{1}H NMR (400 MHz, DMSO-d_6) δ12.21 (s, 1H), 8.25 (s, 1H), 8.06-8.00 (m, 1H), 7.87 (s, 1H), 7.17 (s, 1H), 6.19 (d, *J* = 7.2 Hz, 1H), 2.10-2.00 (m, 1H), 1.19-1.12 (m, 2H), 0.93-0.87 (m, 2H). LCMS (ESI/APCI) m/z: 273.8 $[M+H]^+$.

Zharp-99-8: 8-chloro-3-cyclopropylthieno[2,3-*g*]quinoline 1,1-dioxide

Intermediate **Zharp-99-7** (200 mg, 0.73 mmol) was added to phosphorus oxychloride (5 mL) and then the mixture was stirred at reflux for 2 hours to afford a light brown solution. After cooling to room temperature, the excess phosphorus oxychloride was removed in vacuum. The remaining residue was added to ethyl acetate (20 mL) with an ice bath. Then, it was slowly neutralized with saturated $NaHCO_3$ aqueous solution. The aqueous layer was extracted with ethyl acetate (20 mL * 3). The combined organic layer was washed with brine, dried over Na_2SO_4 and concentrated to give the desired product (200 mg, 94%) as a grey solid. ^{1}H NMR (400 MHz, DMSO-d_6) δ 9.06 (d, *J* = 4.0 Hz, 1H), 8.76 (s, 1H), 8.36 (s, 1H), 8.16 (s, 1H), 8.01 (d, *J* = 4.0 Hz, 1H).

Zharp-99: 8-(benzo[*d*]thiazol-5-ylamino)-3-cyclopropylthieno[2,3-*g*]quinoline 1,1-dioxide

To a solution of **Zharp-99-8** (120 mg, 0.41 mmol) in isopropanol (15 mL) was added benzo[*d*]thiazol-5-amine (74 mg, 0.49 mmol) and conc. HCl (1 drop). The mixture was stirred at reflux for 1 hour. After cooling to room temperature, the solid was precipitated from the solution. The solid was collected via filtration, washed with ethanol (2 mL) and dried in vacuum to give the desired product **Zharp-99** (98 mg, 51%) as a yellow solid. ^{1}H NMR (400 MHz, DMSO-d_6) δ 15.10 (s, 1H), 11.35 (s, 1H), 9.54 (s, 1H), 9.35 (s, 1H), 8.58 (d, *J* = 6.8 Hz, 1H), 8.46 – 8.31 (m, 2H), 8.24 (s, 1H), 7.63 (d, *J* = 8.4 Hz, 1H), 7.41 (s, 1H), 7.00 (d, *J* = 6.8 Hz, 1H), 2.19 – 2.07 (m, 1H), 1.31 – 1.18 (m, 2H), 1.01 – 0.93 (m, 2H). ^{13}C NMR (151 MHz, DMSO-d_6) δ 157.5, 154.1, 153.2, 151.5,

150.8, 149.4, 138.5, 133.6, 132.5, 129.4, 123.9, 123.3, 122.9, 121.5, 119.3, 116.7, 116.4, 103.2, 39.9, 39.8, 39.7, 39.5, 39.4, 39.2, 39.1, 9.9, 8.2. HRMS (ESI) calcd for $C_{21}H_{16}N_3O_2S_2$ $[M + H]^+$, 406.0678; found, 406.0678. Purity: 99.8%.
